# Crystal structure and Hirshfeld surface analysis of (*Z*)-4-oxo-4-{phen­yl[(thio­phen-2-yl)meth­yl]amino}­but-2-enoic acid

**DOI:** 10.1107/S2056989024003967

**Published:** 2024-05-10

**Authors:** Alexandra G. Podrezova, Eugeniya V. Nikitina, Mikhail S. Grigoriev, Mehmet Akkurt, Khudayar I. Hasanov, Nurlana D. Sadikhova, Ajaya Bhattarai

**Affiliations:** a RUDN University, 6 Miklukho-Maklaya St., Moscow 117198, Russian Federation; b A. N. Frumkin Institute of Physical Chemistry and Electrochemistry, Russian Academy of Sciences, Leninsky pr. 31, bld. 4, Moscow, Russian Federation; c Department of Physics, Faculty of Sciences, Erciyes University, 38039 Kayseri, Türkiye; d Azerbaijan Medical University, Scientific Research Centre (SRC), A. Kasumzade St. 14. AZ 1022, Baku, Azerbaijan; e Western Caspian University, Istiqlaliyyat Street 31, AZ1001, Baku, Azerbaijan; f Department of Chemistry, Baku State University, Z. Xalilov Str. 23, Az 1148 Baku, Azerbaijan; g Department of Chemistry, M.M.A.M.C (Tribhuvan University), Biratnagar, Nepal; Texas A & M University, USA

**Keywords:** crystal structure, thio­phene ring, hydrogen bonds, disorder, Hirshfeld surface analysis

## Abstract

In the title compound, C_15_H_13_NO_3_S, the mol­ecular conformation is stable with the intra­molecular O—H⋯O hydrogen bond forming a *S*(7) ring motif. In the crystal, mol­ecules are connected by C—H⋯O hydrogen bonds, forming *C*(8) chains running along the *a*-axis direction. Cohesion of the packing is provided by weak van der Waals inter­actions between the chains. A Hirshfeld surface analysis was undertaken to investigate and qu­antify the inter­molecular inter­actions. The thio­phene ring is disordered in a 0.9466 (17):0.0534 (17) ratio over two positions rotated by 180°.

## Abstract 2

In the crystal, mol­ecules are connected by C—H⋯O hydrogen bonds, forming *C*(8) chains running along the *a*-axis direction. Cohesion of the packing is provided by weak van der Waals inter­actions between the chains.

## 1. Chemical context

**Figure 1:**
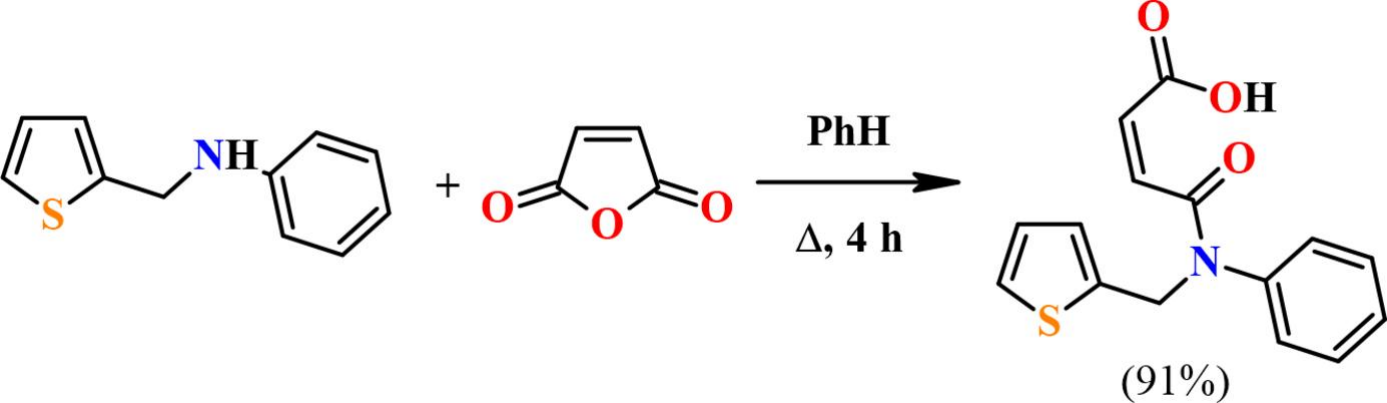
Preparation of the title compound.

The heterocyclic moiety of thio­phene makes it a versatile building block for pharmaceuticals, polymers, and advanced materials (Abdelhamid *et al.*, 2011 ▸; Chawla *et al.*, 2023 ▸; Chan & Ng, 1998 ▸; Khalilov *et al.*, 2021 ▸; Safavora *et al.*, 2019 ▸). One of the inter­esting synthetic directions for thio­phene is its introduction into the Diels–Alder reaction. Highly aromatic thio­phene cannot undergo thermal or catalytic Diels–Alder reactions at normal pressure; special conditions are therefore required to fully unlock its synthetic potential in concerted cyclo­addition reactions (Rulev & Zubkov, 2022 ▸; Polyanskii *et al.*, 2019 ▸). The first Diels–Alder adduct between thio­phene and maleic anhydride, together with some of the simplest dienophiles, was synthesized under 17 kbar pressure and at almost room temperature (Kotsuki *et al.*, 1978 ▸; McCluskey *et al.*, 2002 ▸; Kumamoto *et al.*, 2004 ▸). Not only the thio­phene moiety, but also its combination with other functional groups such as –COOH and C=O can be used as a synthetic strategy for the design of new catalysts, sensors or analytical reagents, and building blocks in crystal engineering (Kopylovich *et al.*, 2011 ▸, 2012*a* ▸,*b* ▸; MacLeod *et al.*, 2012 ▸; Mahmoudi *et al.*, 2017*a* ▸,*b* ▸; Mahmudov *et al.*, 2010 ▸, 2011 ▸; Martins *et al.*, 2017 ▸). The attached substituents can also participate in weak inter­molecular inter­actions to direct the functional properties of new thio­phene derivatives (Maharramov *et al.*, 2010 ▸; Mahmoudi *et al.*, 2019 ▸, 2021 ▸; Shikhaliyev *et al.*, 2019 ▸; Velásquez *et al.*, 2019 ▸). To further investigate the potential of thio­phene derivatives as dienophiles in Diels–Alder reactions (see Krishna *et al.*, 2022 ▸), the title compound **1** was specifically designed and synthesized. The present work showcases a facile methodology for the synthesis of compound **1** from a thio­phene derivative and maleic anhydride – the title compound was isolated in a 91% yield after a standard treatment of the reaction mixture (Fig. 1 ▸). 
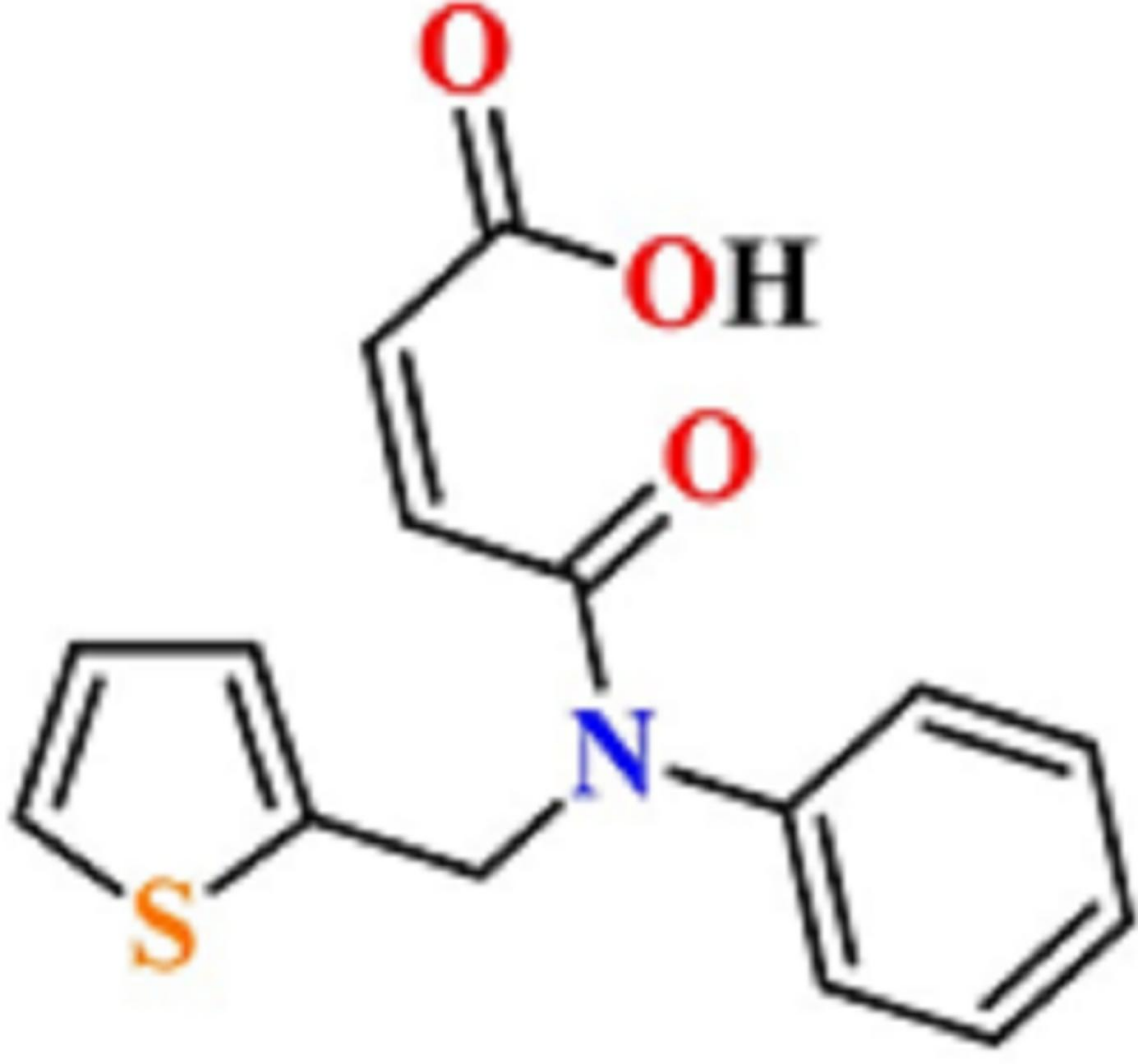


## 2. Structural commentary

**Figure 2:**
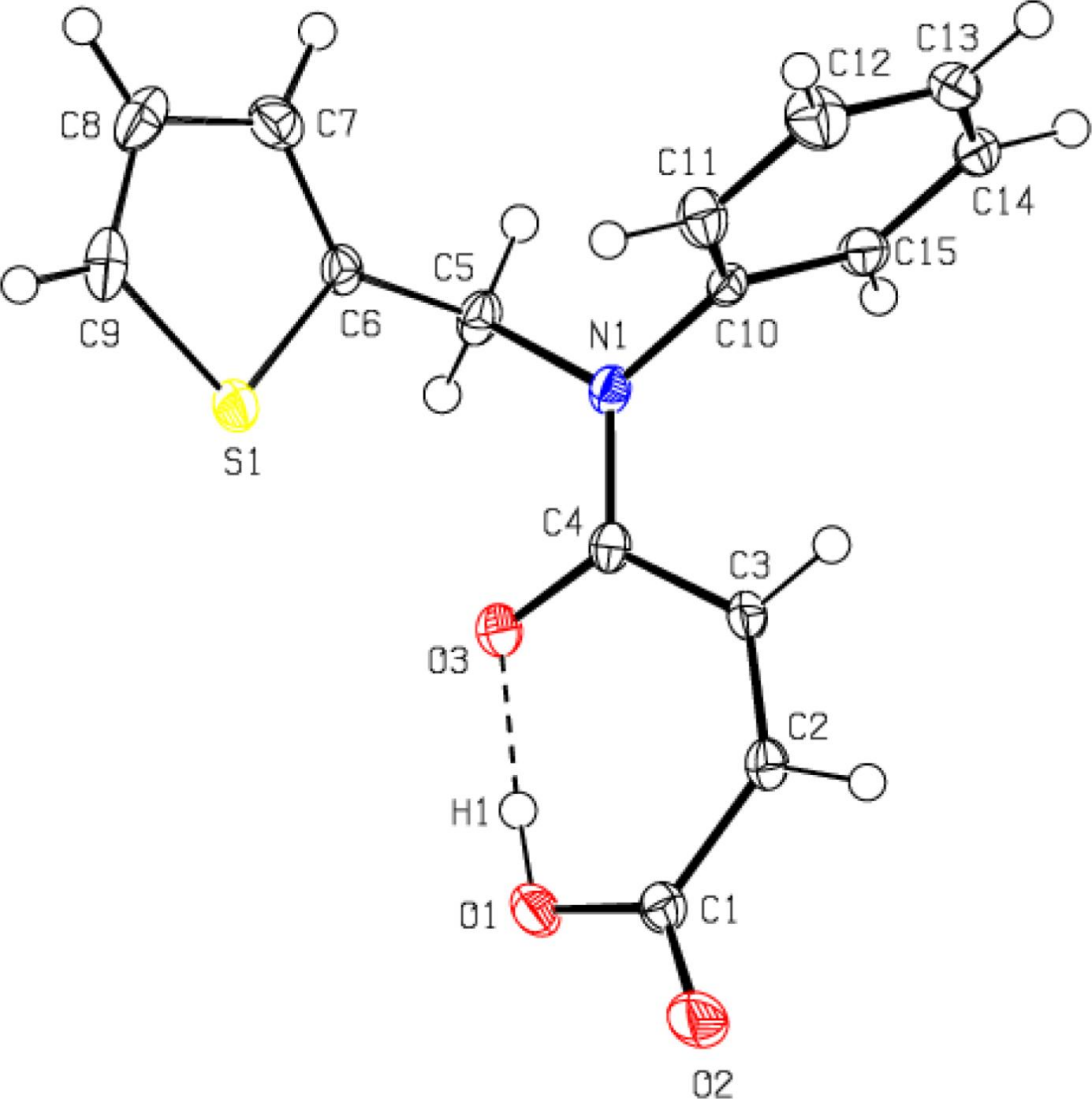
Mol­ecular structure of the title compound showing the atom labelling and ellipsoids at the 50% probability level. Only the major disordered component is shown.

The mol­ecular conformation of the title compound (Fig. 2 ▸) is stabilized by the intra­molecular O—H⋯O hydrogen bond, which forms an *S*(7) ring motif (Bernstein *et al.*, 1995 ▸; Table 1 ▸). The thio­phene ring (S1/C6–C9) is disordered in a 0.9466 (17):0.0534 (17) ratio over two positions rotated by 180°. The phenyl ring (C10–C15) makes dihedral angles of 62.45 (8) and 63.1 (5)°, respectively, with the major and minor disorder components (S1/C6–C9 and S1*A*/C6*A*–C9*A*) of the thio­phene ring. The sum of the angles around N1 of 359.9° is typical for secondary amides. The N1—C4—C3—C2, C4—C3—C2—C1, C3—C2—C1—O1 and C3—C2—C1—O2 torsion angles are 172.40 (13), −3.2 (2), 14.4 (2) and −165.63 (15)°, respectively. The bond lengths and angles in the title compound are comparable to those of the similar compounds reported in the *Database survey*.

## 3. Supra­molecular features and Hirshfeld surface analysis

**Figure 3:**
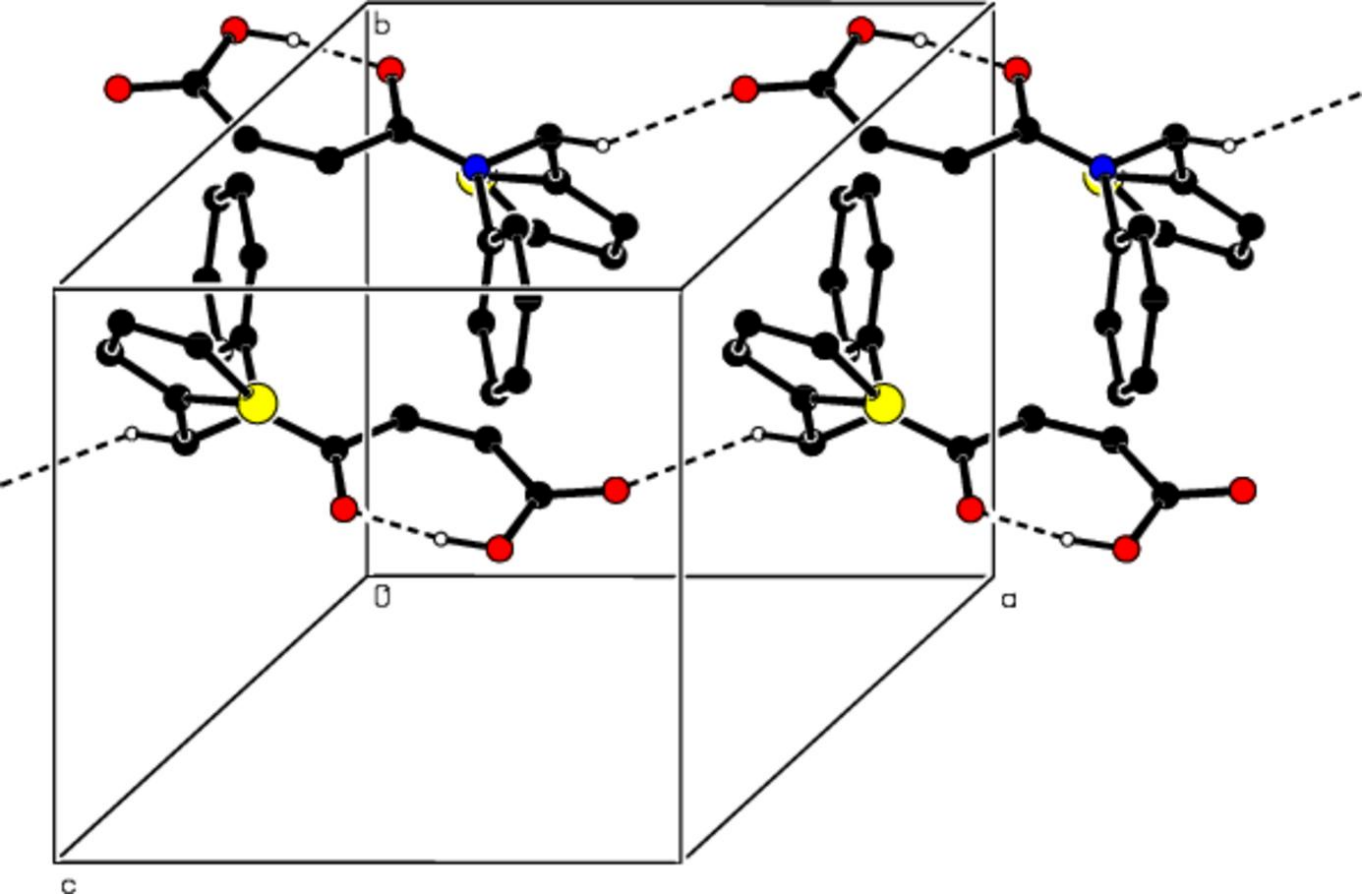
Crystal packing showing the chains along the *a*-axis direction formed by C—H⋯O and O—H⋯O hydrogen bonds (dashed lines). For clarity, the minor disordered component and H atoms not involved in hydrogen bonding were omitted.

In the crystal, mol­ecules are connected by C—H⋯O hydrogen bonds, forming *C*(8) chains running along the *a*-axis direction (Table 1 ▸; Figs. 3 ▸ and 4 ▸). Cohesion of the packing is provided by weak van der Waals inter­actions between the chains.

**Figure 5:**
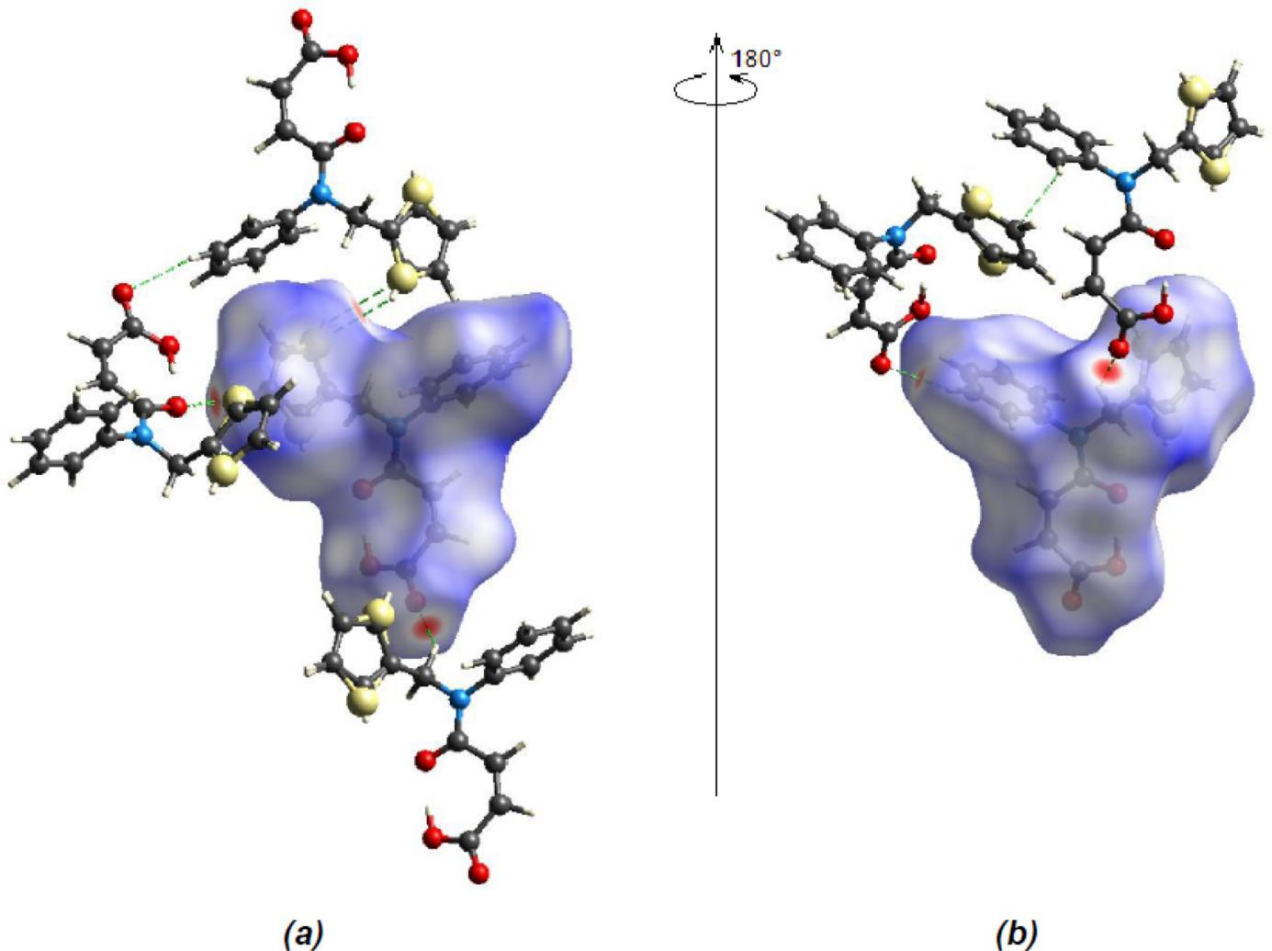
Front (*a*) and back (*b*) views of the three-dimensional Hirshfeld surface, with some C—H⋯O and O—H⋯O hydrogen bonds shown as dashed lines.

A Hirshfeld surface analysis was performed to further investigate the inter­molecular inter­actions present in the title compound and the two-dimensional fingerprint plots were generated with *CrystalExplorer17.5* (Spackman *et al.*, 2021 ▸). Fig. 5 ▸ shows the three-dimensional Hirshfeld surface of the compound with *d*_norm_ (normalized contact distance) plotted over the range −0.2048 (red) to +1.3169 (blue) a.u.

**Figure 6:**
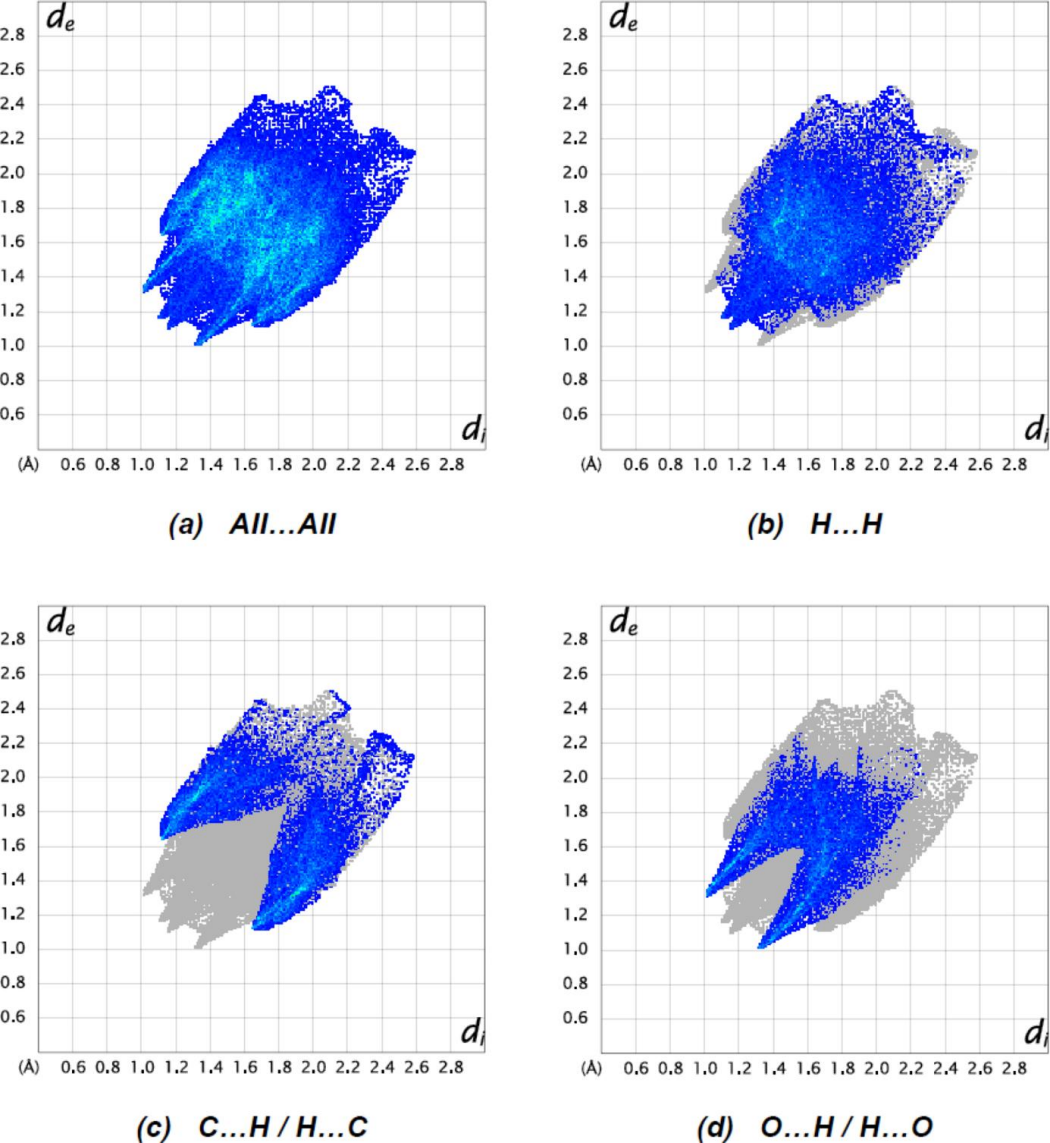
The two-dimensional fingerprint plots for the title mol­ecule showing (*a*) all inter­actions, and delineated into (*b*) H⋯H, (*c*) C⋯H/H⋯C and (*d*) O⋯H/H⋯O inter­actions. The *d*_i_ and *d*_e_ values are the closest inter­nal and external distances (in Å) from given points on the Hirshfeld surface.

The fingerprint plots (Fig. 6 ▸) show that H⋯H [Fig. 6 ▸(*b*); 43.2%], C⋯H/H⋯C [Fig. 6 ▸(*c*); 27.7%] and O⋯H/H⋯O [Fig. 6 ▸(*d*); 23.7%] inter­actions contribute the most to the surface contacts. The percentage contributions to the Hirshfeld surfaces from other minor inter­atomic contacts are as follows: S⋯H/H⋯S 2.5%, C⋯O/O⋯C 1.0%, O⋯O 0.8%, C⋯C 0.5%, N⋯O/O⋯N 0.3% and S⋯O/O⋯S 0.2%.

## 4. Database survey

A search of the Cambridge Structural Database (CSD, version 5.43, update of June 2022; Groom *et al.*, 2016 ▸) for the *N-[(thio­phen-2-yl)meth­yl]aniline* unit gave two similar structures, *viz.* 3,4-dimethyl-*N*-[1-(1-thio­phen-2-yl)ethyl­idene]aniline (CSD refcode VIKXIY: Su *et al.*, 2013 ▸) and *N*-((*E*)-{5-[(*E*)-(pyridin-3-yl­imino)­meth­yl]thio­phen-2-yl}methyl­idene)pyridin-3-amine (QIQLAF: Bolduc *et al.*, 2013 ▸). In VIKXIY, mol­ecules are linked by non-classical C—H⋯N hydrogen bonds into supra­molecular chains. The three-dimensional network of QIQLAF is governed by multiple weak inter­actions, including π-stacking between inter­calated thio­phene rings and azomethine bonds. The mol­ecules are oriented in *anti* or *syn* orientations as a result of the hydrogen-bonding inter­actions in the crystal.

## 5. Synthesis and crystallization

A mixture of *N*-(thio­phen-2-ylmeth­yl)aniline (1.89 g, 10 mmol) and maleic anhydride (0.98 g, 10 mmol) was refluxed for 4 h in benzene (20 mL) (TLC monitoring). The reaction mixture was then concentrated under reduced pressure and the obtained slightly yellow oil was solidified in hexane. The solid was recrystallized from a mixture of hexa­ne/ethyl acetate (*v*/*v* ∼5:4). The title compound was obtained as colourless prisms (2.61 g, 9.09 mmol). Yield 91%, m.p. 374.3–375.8 K. Single crystals were grown from a mixture of hexa­ne/ethyl acetate (∼5:4). ^1^H NMR (700 MHz, DMSO-*d*_6_) (*J*, Hz): *δ* 12.75 (*s*, 1H), 7.41–7.37 (*m*, 1H), 7.36–7.31 (*m*, 2H), 7.31–7.26 (*m*, 1H), 7.18 (*d*, *J* = 7.3 Hz, 2H), 6.92–6.87 (*m*, 2H), 6.38 (*d*, *J* = 12.0, 1H), 5.78 (*d*, *J* = 12.0, 1H), 5.07 (*s*, 2H). ^13^C NMR (175 MHz, CDCl_3_): *δ* 165.87, 164.96, 139.69, 136.67, 136.36, 130.31, 129.63, 128.60, 128.50, 127.79, 126.78, 126.70, 48.81. HRMS (ESI–TOF): calculated for C_15_H_13_NO_3_S [M + H]^+^ 288.0694; found 288.0691. Elemental analysis calculated (%) for C_15_H_13_NO_3_S: C 62.70; H, 4.56; N, 4.87; O, 16.70; S, 11.16; found: C 62.82; H, 4.45; N, 4.94; O, 16.74; S, 11.04.

## 6. Refinement

Crystal data, data collection and structure refinement details are summarized in Table 2 ▸. The thio­phene ring (S1/C6–C9) is disordered in a ratio of 0.9466 (17): 0.0534 (17) over two positions with a rotation of 180°. C-bound H atoms were placed in their geometrically calculated positions and refined using a riding model, with C—H = 0.95–0.99 Å and *U*_iso_(H) = 1.2*U*_eq_(C) for aromatic and methyl­ene H atoms. The H atom of the OH group was found in a difference-Fourier map and refined freely.

## Supplementary Material

Crystal structure: contains datablock(s) I. DOI: 10.1107/S2056989024003967/jy2046sup1.cif

Structure factors: contains datablock(s) I. DOI: 10.1107/S2056989024003967/jy2046Isup2.hkl

Supporting information file. DOI: 10.1107/S2056989024003967/jy2046Isup3.cml

CCDC reference: 2352378

Additional supporting information:  crystallographic information; 3D view; checkCIF report

## Figures and Tables

**Figure 4:**
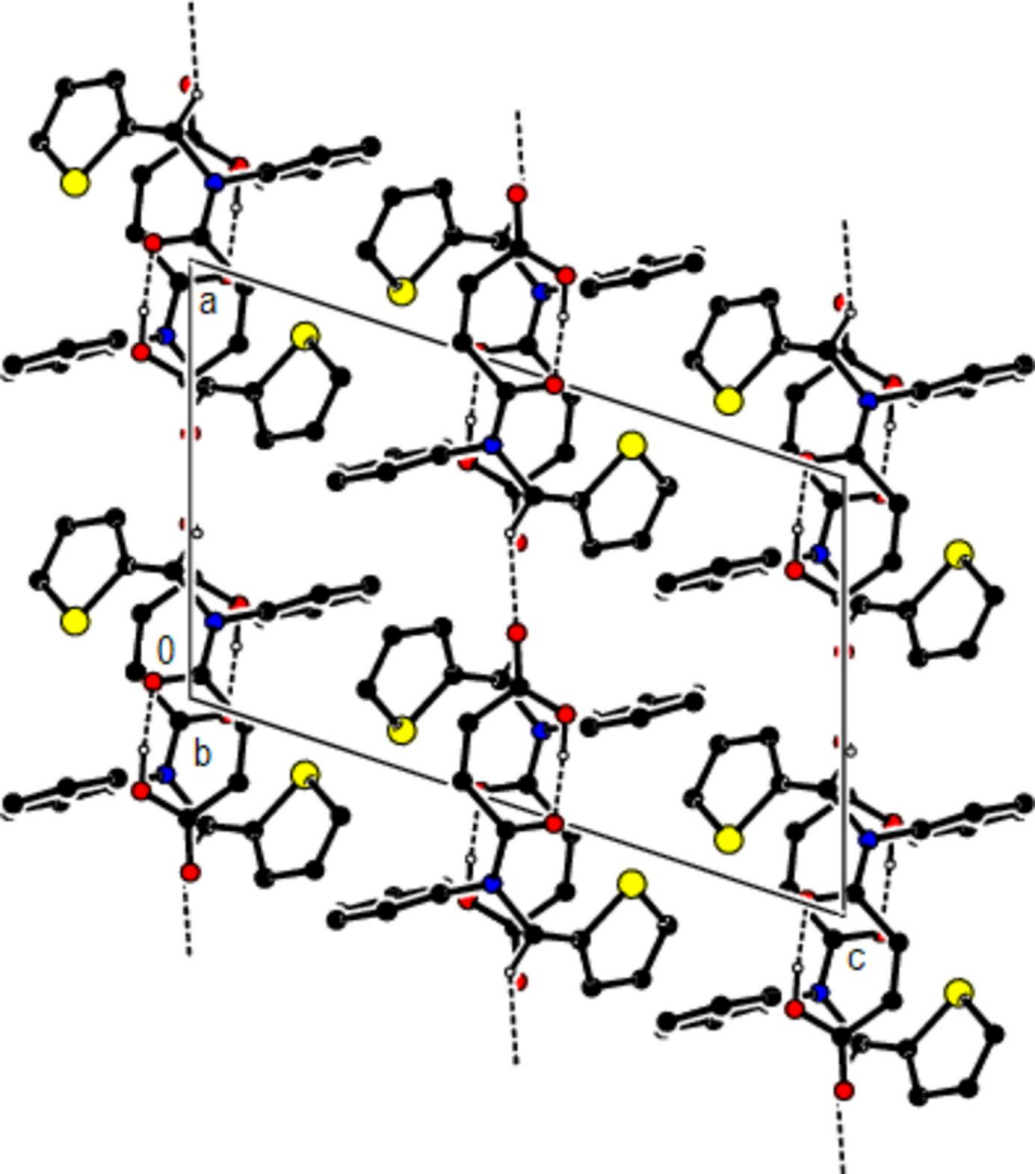
Crystal packing along the *b* axis showing C—H⋯O and O—H⋯O hydrogen bonds (dashed lines).

**Table 1:** Hydrogen-bond geometry (Å, °)

*D*—H⋯*A*	*D*—H	H⋯*A*	*D*⋯*A*	*D*—H⋯*A*
O1—H1⋯O3	0.93 (3)	1.59 (3)	2.5153 (15)	172 (2)
C5—H5*B*⋯O2^i^	0.99	2.40	3.2201 (19)	140

Symmetry code: (i) 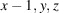 .

**Table 2:** Experimental details

Crystal data	
Chemical formula	C_15_H_13_NO_3_S
*M*_r_	287.32
Crystal system, space group	Monoclinic, *P*2_1_/*c*
Temperature (K)	100
*a*, *b*, *c* (Å)	9.8042 (8), 9.4258 (8), 15.4065 (13)
β (°)	108.455 (3)
*V* (Å^3^)	1350.5 (2)
*Z*	4
Radiation type	Mo *K*α
μ (mm^−1^)	0.25
Crystal size (mm)	0.40 × 0.34 × 0.20

Data collection	
Diffractometer	Bruker Kappa APEXII area-detector diffractometer
Absorption correction	Multi-scan (*SADABS*; Krause *et al.*, 2015 ▸)
*T*_min_, *T*_max_	0.940, 1.000
No. of measured, independent and observed [*I* > 2σ(*I*)] reflections	21582, 4012, 3177
*R*_int_	0.043
(sin θ/λ)_max_ (Å^−1^)	0.708

Refinement	
*R*[*F* ^2^ > 2σ(*F* ^2^)], *wR*(*F* ^2^), *S*	0.038, 0.097, 1.03
No. of reflections	4012
No. of parameters	192
No. of restraints	12
H-atom treatment	H atoms treated by a mixture of independent and constrained refinement
Δρ_max_, Δρ_min_ (e Å^−3^)	0.35, −0.34

Computer programs: *APEX4* and *SAINT* (Bruker, 2018 ▸), *SHELXT* (Sheldrick, 2015*a* ▸), *SHELXL2018* (Sheldrick, 2015*b* ▸), *ORTEP-3 for Windows* (Farrugia, 2012 ▸) and *PLATON* (Spek, 2020 ▸).

## supplementary crystallographic information

### Crystal data

**Table:**

C_15_H_13_NO_3_S	*F*(000) = 600
*M**_r_* = 287.32	*D*_x_ = 1.413 Mg m^−^^3^
Monoclinic, *P*2_1_/*c*	Mo *K*α radiation, λ = 0.71073 Å
*a* = 9.8042 (8) Å	Cell parameters from 4604 reflections
*b* = 9.4258 (8) Å	θ = 2.8–29.0°
*c* = 15.4065 (13) Å	µ = 0.25 mm^−^^1^
β = 108.455 (3)°	*T* = 100 K
*V* = 1350.5 (2) Å^3^	Bulk, colourless
*Z* = 4	0.40 × 0.34 × 0.20 mm

### Data collection

**Table:**

Bruker Kappa APEXII area-detector diffractometer	3177 reflections with *I* > 2σ(*I*)
φ and ω scans	*R*_int_ = 0.043
Absorption correction: multi-scan (SADABS; Krause *et al.*, 2015)	θ_max_ = 30.2°, θ_min_ = 3.0°
*T*_min_ = 0.940, *T*_max_ = 1.000	*h* = −13→13
21582 measured reflections	*k* = −13→13
4012 independent reflections	*l* = −21→21

### Refinement

**Table:**

Refinement on *F*^2^	12 restraints
Least-squares matrix: full	Hydrogen site location: mixed
*R*[*F*^2^ > 2σ(*F*^2^)] = 0.038	H atoms treated by a mixture of independent and constrained refinement
*wR*(*F*^2^) = 0.097	*w* = 1/[σ^2^(*F*_o_^2^) + (0.0437*P*)^2^ + 0.4392*P*] where *P* = (*F*_o_^2^ + 2*F*_c_^2^)/3
*S* = 1.03	(Δ/σ)_max_ = 0.001
4012 reflections	Δρ_max_ = 0.35 e Å^−^^3^
192 parameters	Δρ_min_ = −0.34 e Å^−^^3^

### Special details

**Table:**

Geometry. All esds (except the esd in the dihedral angle between two l.s. planes) are estimated using the full covariance matrix. The cell esds are taken into account individually in the estimation of esds in distances, angles and torsion angles; correlations between esds in cell parameters are only used when they are defined by crystal symmetry. An approximate (isotropic) treatment of cell esds is used for estimating esds involving l.s. planes.

### Fractional atomic coordinates and isotropic or equivalent isotropic displacement parameters (Å^2^)

**Table:**

	*x*	*y*	*z*	*U*_iso_*/*U*_eq_	Occ. (<1)
C1	1.27590 (14)	0.14305 (14)	0.00355 (9)	0.0182 (3)	
C2	1.15520 (14)	0.20128 (14)	−0.07416 (8)	0.0170 (3)	
H2	1.180897	0.219090	−0.127661	0.020*	
C3	1.01839 (14)	0.23331 (14)	−0.08348 (8)	0.0158 (2)	
H3	0.964652	0.274345	−0.140347	0.019*	
C4	0.93968 (14)	0.21300 (13)	−0.01641 (8)	0.0148 (2)	
C5	0.72253 (14)	0.24616 (15)	0.02527 (9)	0.0182 (3)	
H5A	0.748802	0.153057	0.055723	0.022*	
H5B	0.619115	0.242997	−0.010667	0.022*	
C6	0.74604 (14)	0.35914 (14)	0.09688 (8)	0.0158 (2)	0.9466 (17)
C7	0.6467 (2)	0.4469 (3)	0.11431 (18)	0.0227 (5)	0.9466 (17)
H7	0.548211	0.447362	0.078155	0.027*	0.9466 (17)
C8	0.70285 (18)	0.53646 (15)	0.19035 (10)	0.0260 (3)	0.9466 (17)
H8	0.647063	0.602554	0.211255	0.031*	0.9466 (17)
C9	0.84581 (17)	0.51709 (16)	0.23009 (9)	0.0247 (3)	0.9466 (17)
H9	0.902561	0.568587	0.281969	0.030*	0.9466 (17)
S1	0.91239 (4)	0.38895 (4)	0.17591 (2)	0.01947 (12)	0.9466 (17)
C6A	0.74604 (14)	0.35914 (14)	0.09688 (8)	0.0158 (2)	0.0534 (17)
C7A	0.866 (2)	0.409 (3)	0.1685 (14)	0.0227 (5)	0.0534 (17)
H7A	0.959121	0.372099	0.176104	0.027*	0.0534 (17)
C8A	0.84581 (17)	0.51709 (16)	0.23009 (9)	0.0247 (3)	0.0534 (17)
H8A	0.912101	0.560641	0.282056	0.030*	0.0534 (17)
C9A	0.70285 (18)	0.53646 (15)	0.19035 (10)	0.0260 (3)	0.0534 (17)
H9A	0.654641	0.604979	0.215253	0.031*	0.0534 (17)
S1A	0.6147 (12)	0.4493 (16)	0.1046 (9)	0.01947 (12)	0.0534 (17)
C10	0.74377 (13)	0.35644 (14)	−0.11738 (8)	0.0145 (2)	
C11	0.76237 (15)	0.50178 (15)	−0.11112 (9)	0.0218 (3)	
H11	0.815581	0.544184	−0.054609	0.026*	
C12	0.70262 (17)	0.58569 (16)	−0.18818 (11)	0.0281 (3)	
H12	0.714737	0.685746	−0.184435	0.034*	
C13	0.62523 (15)	0.52269 (17)	−0.27055 (10)	0.0264 (3)	
H13	0.585703	0.579641	−0.323445	0.032*	
C14	0.60560 (15)	0.37755 (17)	−0.27577 (9)	0.0248 (3)	
H14	0.551520	0.335301	−0.332142	0.030*	
C15	0.66448 (14)	0.29248 (16)	−0.19901 (9)	0.0205 (3)	
H15	0.650653	0.192624	−0.202446	0.025*	
N1	0.80705 (11)	0.26905 (12)	−0.03776 (7)	0.0152 (2)	
O1	1.24869 (11)	0.08523 (11)	0.07469 (6)	0.0217 (2)	
H1	1.153 (3)	0.100 (2)	0.0707 (15)	0.065 (7)*	
O2	1.39697 (11)	0.14995 (13)	−0.00031 (7)	0.0295 (3)	
O3	0.99128 (10)	0.14570 (10)	0.05663 (6)	0.0200 (2)	

### Atomic displacement parameters (Å^2^)

**Table:**

	*U*^11^	*U*^22^	*U*^33^	*U*^12^	*U*^13^	*U*^23^
C1	0.0177 (6)	0.0198 (6)	0.0159 (6)	−0.0010 (5)	0.0038 (5)	−0.0025 (5)
C2	0.0193 (6)	0.0189 (6)	0.0136 (6)	−0.0008 (5)	0.0066 (5)	0.0015 (5)
C3	0.0178 (6)	0.0185 (6)	0.0112 (5)	0.0003 (5)	0.0047 (5)	0.0013 (4)
C4	0.0176 (6)	0.0137 (6)	0.0134 (5)	−0.0028 (5)	0.0054 (5)	−0.0022 (4)
C5	0.0175 (6)	0.0233 (7)	0.0168 (6)	−0.0040 (5)	0.0099 (5)	−0.0021 (5)
C6	0.0172 (6)	0.0188 (6)	0.0124 (5)	−0.0013 (5)	0.0062 (5)	0.0018 (4)
C7	0.0191 (11)	0.0285 (9)	0.0199 (10)	0.0011 (9)	0.0052 (9)	0.0066 (7)
C8	0.0398 (9)	0.0198 (7)	0.0266 (7)	0.0030 (6)	0.0223 (7)	0.0014 (5)
C9	0.0383 (8)	0.0224 (7)	0.0158 (6)	−0.0067 (6)	0.0120 (6)	−0.0037 (5)
S1	0.01743 (19)	0.0253 (2)	0.01436 (17)	−0.00051 (14)	0.00317 (13)	−0.00162 (13)
C6A	0.0172 (6)	0.0188 (6)	0.0124 (5)	−0.0013 (5)	0.0062 (5)	0.0018 (4)
C7A	0.0191 (11)	0.0285 (9)	0.0199 (10)	0.0011 (9)	0.0052 (9)	0.0066 (7)
C8A	0.0383 (8)	0.0224 (7)	0.0158 (6)	−0.0067 (6)	0.0120 (6)	−0.0037 (5)
C9A	0.0398 (9)	0.0198 (7)	0.0266 (7)	0.0030 (6)	0.0223 (7)	0.0014 (5)
S1A	0.01743 (19)	0.0253 (2)	0.01436 (17)	−0.00051 (14)	0.00317 (13)	−0.00162 (13)
C10	0.0124 (5)	0.0188 (6)	0.0131 (5)	0.0004 (4)	0.0051 (4)	−0.0020 (4)
C11	0.0256 (7)	0.0202 (7)	0.0179 (6)	−0.0035 (5)	0.0046 (5)	−0.0031 (5)
C12	0.0340 (8)	0.0208 (7)	0.0287 (8)	0.0009 (6)	0.0090 (6)	0.0044 (6)
C13	0.0223 (7)	0.0383 (9)	0.0194 (7)	0.0112 (6)	0.0077 (5)	0.0074 (6)
C14	0.0176 (6)	0.0385 (9)	0.0154 (6)	0.0071 (6)	0.0011 (5)	−0.0072 (6)
C15	0.0168 (6)	0.0228 (7)	0.0192 (6)	0.0020 (5)	0.0020 (5)	−0.0077 (5)
N1	0.0147 (5)	0.0185 (5)	0.0133 (5)	−0.0023 (4)	0.0059 (4)	−0.0017 (4)
O1	0.0193 (5)	0.0284 (5)	0.0159 (4)	0.0020 (4)	0.0033 (4)	0.0047 (4)
O2	0.0152 (5)	0.0472 (7)	0.0249 (5)	0.0013 (5)	0.0047 (4)	0.0033 (5)
O3	0.0229 (5)	0.0231 (5)	0.0155 (4)	0.0028 (4)	0.0080 (4)	0.0045 (3)

### Geometric parameters (Å, º)

**Table:**

C1—O2	1.2089 (16)	C6A—C7A	1.414 (17)
C1—O1	1.3246 (16)	C6A—S1A	1.579 (12)
C1—C2	1.4960 (18)	C7A—C8A	1.447 (18)
C2—C3	1.3375 (18)	C7A—H7A	0.9500
C2—H2	0.9500	C8A—C9A	1.353 (2)
C3—C4	1.4848 (17)	C8A—H8A	0.9500
C3—H3	0.9500	C9A—S1A	1.563 (12)
C4—O3	1.2507 (15)	C9A—H9A	0.9500
C4—N1	1.3444 (16)	C10—C11	1.3813 (19)
C5—N1	1.4783 (16)	C10—C15	1.3895 (17)
C5—C6	1.4978 (18)	C10—N1	1.4444 (16)
C5—C6A	1.4978 (18)	C11—C12	1.392 (2)
C5—H5A	0.9900	C11—H11	0.9500
C5—H5B	0.9900	C12—C13	1.389 (2)
C6—C7	1.368 (3)	C12—H12	0.9500
C6—S1	1.7217 (13)	C13—C14	1.380 (2)
C7—C8	1.407 (3)	C13—H13	0.9500
C7—H7	0.9500	C14—C15	1.394 (2)
C8—C9	1.353 (2)	C14—H14	0.9500
C8—H8	0.9500	C15—H15	0.9500
C9—S1	1.7108 (15)	O1—H1	0.93 (3)
C9—H9	0.9500		
			
O2—C1—O1	121.40 (12)	C5—C6A—S1A	120.0 (4)
O2—C1—C2	118.76 (12)	C6A—C7A—C8A	119.6 (16)
O1—C1—C2	119.85 (12)	C6A—C7A—H7A	120.2
C3—C2—C1	132.93 (12)	C8A—C7A—H7A	120.2
C3—C2—H2	113.5	C9A—C8A—C7A	97.8 (8)
C1—C2—H2	113.5	C9A—C8A—H8A	131.1
C2—C3—C4	128.36 (12)	C7A—C8A—H8A	131.1
C2—C3—H3	115.8	C8A—C9A—S1A	121.9 (4)
C4—C3—H3	115.8	C8A—C9A—H9A	119.1
O3—C4—N1	120.25 (11)	S1A—C9A—H9A	119.1
O3—C4—C3	122.70 (12)	C9A—S1A—C6A	95.8 (6)
N1—C4—C3	117.04 (11)	C11—C10—C15	121.14 (12)
N1—C5—C6	113.11 (10)	C11—C10—N1	119.64 (11)
N1—C5—C6A	113.11 (10)	C15—C10—N1	119.22 (12)
N1—C5—H5A	109.0	C10—C11—C12	119.57 (13)
C6—C5—H5A	109.0	C10—C11—H11	120.2
N1—C5—H5B	109.0	C12—C11—H11	120.2
C6—C5—H5B	109.0	C13—C12—C11	119.80 (14)
H5A—C5—H5B	107.8	C13—C12—H12	120.1
C7—C6—C5	128.44 (15)	C11—C12—H12	120.1
C7—C6—S1	109.66 (13)	C14—C13—C12	120.18 (13)
C5—C6—S1	121.83 (10)	C14—C13—H13	119.9
C6—C7—C8	114.29 (17)	C12—C13—H13	119.9
C6—C7—H7	122.9	C13—C14—C15	120.54 (13)
C8—C7—H7	122.9	C13—C14—H14	119.7
C9—C8—C7	111.84 (15)	C15—C14—H14	119.7
C9—C8—H8	124.1	C10—C15—C14	118.76 (13)
C7—C8—H8	124.1	C10—C15—H15	120.6
C8—C9—S1	112.04 (11)	C14—C15—H15	120.6
C8—C9—H9	124.0	C4—N1—C10	123.64 (10)
S1—C9—H9	124.0	C4—N1—C5	118.81 (11)
C9—S1—C6	92.17 (7)	C10—N1—C5	117.49 (10)
C7A—C6A—C5	135.0 (9)	C1—O1—H1	110.4 (14)
C7A—C6A—S1A	104.9 (9)		
			
O2—C1—C2—C3	−165.63 (15)	C5—C6A—S1A—C9A	176.9 (3)
O1—C1—C2—C3	14.4 (2)	C15—C10—C11—C12	−1.0 (2)
C1—C2—C3—C4	−3.2 (2)	N1—C10—C11—C12	179.31 (12)
C2—C3—C4—O3	−8.5 (2)	C10—C11—C12—C13	−0.1 (2)
C2—C3—C4—N1	172.40 (13)	C11—C12—C13—C14	1.0 (2)
N1—C5—C6—C7	121.71 (19)	C12—C13—C14—C15	−0.8 (2)
N1—C5—C6—S1	−61.84 (14)	C11—C10—C15—C14	1.2 (2)
C5—C6—C7—C8	176.46 (15)	N1—C10—C15—C14	−179.09 (11)
S1—C6—C7—C8	−0.3 (2)	C13—C14—C15—C10	−0.3 (2)
C6—C7—C8—C9	0.6 (3)	O3—C4—N1—C10	175.49 (11)
C7—C8—C9—S1	−0.54 (19)	C3—C4—N1—C10	−5.36 (17)
C8—C9—S1—C6	0.30 (12)	O3—C4—N1—C5	−1.68 (17)
C7—C6—S1—C9	0.02 (15)	C3—C4—N1—C5	177.47 (11)
C5—C6—S1—C9	−177.02 (11)	C11—C10—N1—C4	−90.21 (15)
N1—C5—C6A—C7A	−61.1 (16)	C15—C10—N1—C4	90.09 (15)
N1—C5—C6A—S1A	121.4 (7)	C11—C10—N1—C5	87.00 (15)
C5—C6A—C7A—C8A	−175.6 (8)	C15—C10—N1—C5	−92.71 (14)
S1A—C6A—C7A—C8A	2 (2)	C6—C5—N1—C4	89.91 (14)
C6A—C7A—C8A—C9A	−2 (2)	C6A—C5—N1—C4	89.91 (14)
C7A—C8A—C9A—S1A	0.9 (13)	C6—C5—N1—C10	−87.43 (13)
C8A—C9A—S1A—C6A	0.2 (10)	C6A—C5—N1—C10	−87.43 (13)
C7A—C6A—S1A—C9A	−1.3 (15)		

### Hydrogen-bond geometry (Å, º)

**Table:**

*D*—H···*A*	*D*—H	H···*A*	*D*···*A*	*D*—H···*A*
O1—H1···O3	0.93 (3)	1.59 (3)	2.5153 (15)	172 (2)
C5—H5*B*···O2^i^	0.99	2.40	3.2201 (19)	140
C9—H9···O3^ii^	0.95	2.48	3.3904 (16)	160
C14—H14···O2^iii^	0.95	2.56	3.4261 (17)	152
C15—H15···*Cg*2^iv^	0.95	3.00	3.504 (6)	115

Symmetry codes: (i) *x*−1, *y*, *z*; (ii) −*x*+2, *y*+1/2, −*z*+1/2; (iii) *x*−1, −*y*+1/2, *z*−1/2; (iv) *x*, −*y*−1/2, *z*−3/2.
